# Preventing, treating, and predicting barbering: A fundamental role for biomarkers of oxidative stress in a mouse model of Trichotillomania

**DOI:** 10.1371/journal.pone.0175222

**Published:** 2017-04-20

**Authors:** Giovana de L. T. Vieira, Amy C. Lossie, Donald C. Lay, John S. Radcliffe, Joseph P. Garner

**Affiliations:** 1 Department of Animal Sciences, Purdue University, West Lafayette, Indiana, United States of America; 2 Livestock Behavior Research Unit, Agricultural Research Service, United States Department of Agriculture, West Lafayette, Indiana, United States of America; 3 Department of Comparative Medicine, Stanford University, Stanford, California, United States of America; 4 (By Courtesy) Department of Psychiatry and Behavioral Sciences, Stanford University, Stanford, California, United States of America; University of British Columbia, CANADA

## Abstract

Barbering, where a “barber” mouse plucks hair from its cagemates or itself, is both a spontaneously occurring abnormal behavior in mice and a well validated model of Trichotillomania (TTM). N-Acetylcysteine, (NAC) a cysteine derived food additive, is remarkably effective in treating TTM patients, but its mechanism of action is unknown. Reactive Oxygen Species (ROS), also known as free radicals, form as a natural byproduct of the normal metabolism of oxygen. Under normal circumstances, cells are able to defend themselves against ROS damage with antioxidant pathways. NAC is the precursor to the main antioxidant produced to defend the brain. Therefore, we hypothesized that barbering is a disease of oxidative stress, whereby ROS and/or a failure of antioxidant defenses leads to neuronal damage that induces barbering in susceptible animals. We tested this hypothesis in 32 female C57BL/6J mice by treating half with 1g/kg BW/day of NAC in their diet, and testing for protection against developing barbering behavior and curing of barbering behavior, and simultaneously testing for a panel of biomarkers of oxidative stress. NAC reduced the chance that mice would be barbers, and this effect did not differ between healthy (i.e. prevention) and affected animals (i.e. cure). Barbering animals had elevated urinary antioxidant capacity, indicative of oxidative stress, at all timepoints. Additionally, after treatment the risk of barbering increased with decreasing hydroxy-2′-deoxyguanosine (8-OHdG) levels, and with increasing glutathione (GSH) and oxidized glutathione (GSSG) levels, further indicating that barbering mice were under oxidative stress regardless of treatment with NAC. We did not find compelling evidence that urinary total antioxidant capacity, or urinary 8-OHdG, could predict response to NAC treatment. We conclude that NAC is effective in preventing and/or curing barbering at least in part by promoting GSH synthesis, thereby preventing oxidative damage.

## Introduction

Trichotillomania (TTM), or human compulsive hair pulling, is one of the most common psychiatric disorders, affecting approximately 3.5% of women, or 3.7 million people in the United States [1]. TTM patients experience pronounced psychological distress with considerable negative impact in their quality of life. Despite its high prevalence and impairment, the etiology and development of the condition are still poorly understood [2].

Barbering behavior in mice consists of hair and/or whisker plucking by itself or the cagemates [3,4] is a well validated animal model of TTM [5]. Despite being highly common in the laboratory setting, little is known about its underlying causes [6]. Similar to TTM, barbering is more common in females, reproductive changes affect the severity of the behavior, and onset is associated with sexual maturation (although barbers onset at a later developmental stage than humans) [4]. Barbers show a pattern of neuropsychological biomarkers that match TTM, but are distinct from OCD or autism patients [5]. There is evidence that metabolism plays a role in the development of barbering behavior. For instance, feeding mice with a diet that increases brain serotonin can exacerbate barbering and induce ulcerative dermatitis, a condition similar to skin picking disorder in humans (which is closely related to Trichotillomania)[7].

At the moment, no prescription drugs have proven to be an effective treatment for TTM [8]. However the most effective non-behavioral treatment for TTM is N-acetylcysteine (NAC) [9,10], a compound whose clinical origins are very different from its current use in psychiatry [11]. NAC is known for its antioxidant properties and used mainly for acetaminophen overdose [12,13]. Nevertheless, 56% of TTM patients who received NAC (1200–2400mg/day orally) for 12 weeks had a reduction of symptoms compared with 16% of patients taking placebo [9].

NAC is an acetylated variant and precursor of the amino acid, L-cysteine, which is oxidized to cystine in the brain (cystine is formed by the dimerization of cysteine via a disulphide bond) [14]. Cystine is a substrate of the glutamate-cystine antiporter, or x(c)-system, which exchanges extracellular cystine for intracellular glutamate, consequently regulating extracellular glutamate levels [15]. The cystine uptake by the glutamate-cystine antiporter is a rate-limiting step in the synthesis of glutathione (GSH) [16], the most abundant antioxidant in the cell [13]. Once cystine enters the cell, it is rapidly reduced to cysteine [17], which combines with glutamate and glycine to produce GSH [18,19], which protects cells against reactive oxygen species (ROS).

ROS are formed during normal aerobic glucose metabolism. Because of their unpaired electrons, ROS molecules are unstable and react easily with other molecules [20]. Under physiological conditions, ROS are counterbalanced by a group of defense pathways including GSH, other antioxidants, antioxidant enzymes and proteins. When the defenses are compromised or ROS are produced in excess, oxidative stress results. Because oxidative stress is acute and pulsatile in nature, individuals under oxidative stress over-produce antioxidants to buffer peaks in ROS production. As a result, a simple measure of oxidative stress is blood or urine total antioxidant capacity (TAC), where elevated TAC is indicative of individuals mounting a response to ongoing elevated oxidative stress [21].

Oxygen radicals can modify or damage proteins, lipids and DNA [22], leading to a series of events that deregulate numerous cellular functions [23,24]. When ROS attacks DNA, they damage nucleotides as well as initiate single- and double-stranded DNA breaks, chromosomal deletions and nucleoside modifications [25]. If not properly removed, DNA damage can exert deleterious effects on normal cell physiology, leading to mutagenesis and/or cell death [26]. The most well-known oxidative DNA byproduct is 8-Hydroxy-2′-deoxyguanosine (8-OHdG), which is formed from a hydroxyl radical attack at the C-8 position of a deoxyguanosine residue in DNA [27]. Measurement of levels of 8-OHdG is frequently used in the evaluation of DNA damage by oxidative radicals. However, the interpretation of 8-OHdG results is complex [28]. 8-OHdG is a byproduct of DNA repair in response to oxidative damage. So high 8-OHdG indicates that oxidative stress occurred in a narrow time frame prior to the assay, and that the individual was able to repair DNA. However low 8-OHdG could reflect a lack of oxidative stress or a failure to repair. Thus 8-OHdG it can only be unambiguously interpreted alongside other oxidative stress biomarkers [28].

Increasing evidence suggests that oxidative stress is involved in the pathogenesis of several diseases [29], including many major neurological disorders like Parkinson's, Alzheimer's, Bipolar disorder and Schizophrenia [30,31]. The high metabolic activity of neurons results in a continuously high ROS production, making the brain particularly vulnerable to oxidative damage [29]. NAC effectively protects neurons from oxidative damage both by increasing GSH levels and directly scavenging ROS [11,13].

Oral administration of GSH alone does not adequately restore GSH levels because of its hydrolysis by intestinal and hepatic enzymes [32]. However, oral administration of NAC results in increased plasma cysteine levels, and subsequently increased plasma GSH. Furthermore, NAC successfully penetrates the blood-brain barrier, resulting in increased levels of GSH in the brain [11,33].

Therefore, we hypothesize that the pathogenesis of barbering/TTM involves oxidative stress/damage of neurons, and that NAC is beneficial to TTM patients because of its ability to increase GSH and consequently protect brain cells from oxidative damage. Other authors have suggested that NAC treats TTM via a direct effect on glutamate transmission [9], since it was shown that systemic administration of NAC can increase the activity of the glutamate-cystine antiporter and restore the concentrations of extracellular glutamate in the nucleus accumbens [34,35], consequently blocking the reinstatement of compulsive behavior [36]. By contrast, we propose that the NAC-induced efflux of glutamate via activation of the x(c)-system, provides both the cystine and glutamate required for the synthesis of GSH, which is stimulated under oxidative stress [18]. Although these two explanations are not mutually exclusive, the role of oxidative stress in barbering would be ruled out if barbering or the response to NAC was not associated with markers of oxidative stress.

Therefore our goal was to test this hypothesis. We predicted that barbers would have increased biomarkers of oxidative stress compared to non-barbers, and that NAC would prevent barbering behavior in normal animals and decrease the behavior in affected animals. The identification of biomarkers for barbering will help to predict individual risk for developing barbering/TTM, as well as aid in the identification, diagnosis, and treatment of individuals with the disorder. In addition, we wanted to evaluate whether these biomarkers could predict individual response to NAC. To our knowledge, this study represents the first attempt to analyze the association between compulsive hair-pulling and oxidative damage.

## Materials and methods

### Animals and housing

A total of 32 female C57BL/6J mice from our breeding colony were used in this study. Animals were obtained from The Jackson Laboratories (Bar Harbor, ME, USA). The age of the animals at the beginning of the study ranged between 2–8 months. Animals were housed with siblings (2–3 per cage) in standard laboratory polycarbonate shoebox cages (Alternative Design, Siloam Springs, AR, USA; 12.7 cm high, 419,35 cm^2^ floor area) with aspen shaving bedding (Harlan Laboratories, Madison, WI, USA) and wire cage lids. Mice were given ad libitum access to food [diet described below] and tap water. The mice were kept on a 14:10 light:dark photoperiod (lights on at 06:00 h and off at 20:00 h), at 60 ± 10% relative humidity and room temperature of 20°C.

Adult CD1 mice were housed in a mouse cage and exposed to dirty bedding from the studied animals in that room. These sentinels were serologic tested every 3 months for *Mycoplasma pulmonis*, ectromelia virus, epizootic diarrhea of infant mice (EDIM), lymphocytic choriomeningitis virus (LCMV), mouse hepatitis virus (MHV), murine norovirus (MNV), mouse parvovirus (MPV), minute virus of mice (MVM), pneumonia virus of mice (PVM), reovirus type 3 (Reo3), Sendai virus, Theiler's mouse encephalomyelitis virus (TMEV) and checked for pinworms and fur mites. All housing and procedures associated with this experiment were approved by Purdue’s Institutional Animal Care and Use Committee.

### Experimental design

#### Diets and NAC treatment

Following earlier experiments [7], two dry powdered diets were formulated from purified ingredients (Purdue Animal Sciences Feed Mill): a "control" diet, which was formulated to match the standard laboratory diet (Rodent diet 5008, www.Labdiet.com) and a "barbering" diet, that increases serotonin synthesis and release and induces barbering in a large proportion of individuals [7]. We were concerned that the level of methionine in the “barbering” diet we had used previously might be insufficient (although in previous work we saw no difference from control in growth and bodyweight in animals on this diet). Therefore this diet was supplemented with 0.450% of Potassium Phosphate Monobasic, 0.2% of Limestone, and 0.532% of DL-methionine. As methionine is converted in the body to cysteine, we supplemented the control diet with 0.155% of DL-methionine, so that the sulfur amino-acid content of the two diets was identical. We wanted to use the “barbering” diet to test whether NAC could prevent the development of barbering seen in our earlier work [7]. Accordingly this diet was given exclusively to half of the non-barbering mice, and the control diet to all the barbers and half of the non-barbers. We then supplemented half of the animals with NAC (Nutrabio, Middlesex, NJ, USA) added at 1 g/kg BW/day. This dosage was calculated using the metabolic scaling of the human dosage of 1200 mg/day [9]. NAC supplementation was randomized and balanced across the diet and baseline barbering status. Thus animals were randomized into 2x2 factorial design.

The diets were administered in Rodent Cafe spill proof feeders (OYC International, Andover, MA, USA) and were administered to 14 cages of mice (32 females total). Since we know that the position of the cage in the rack affects the severity of barbering [4], the treatments were assigned to rack position in a balanced randomized fashion using a Latin square.

We had intended to cross-over the NAC treatment after 12 weeks. However at this point it was clear that the additional methionine might be confounding the experiment. For instance the subjective body condition of the mice, including coat gloss, and eye brightness, was markedly different from other animals of equivalent age in the colony. We therefore opted to maintain animals on the same treatment combinations until 24 weeks, and reformulate the diets without the additional methionine i.e. control diet was now equivalent to the standard laboratory mouse diet, and “barber” diet was as reported in [7]. Thus for detailed diet formulations, see [7].

### Procedures

#### Barbering maps

Patterns of hair-loss were recorded and quantified at baseline (before diet administration), and every two weeks until the end of the 24 weeks using "barber-maps" (diagrams of mouse bodies overlaid by a grid) as previously described [4]. Using the deductive procedure outlined in [4] the barber status of each mouse (self-barber, cagemate-barber, or both) was monitored and recorded every two weeks. Barbering has a very characteristic presentation. Thus for hair loss to be considered barbering, the fur-lesion had to be non-pruritic (i.e. the underlying skin was normal, without evidence of redness or inflammation), the fur-lesion could not show signs of aggression or self-injury (i.e. there was no scaring or scabbing on or around the fur lesion, nor could it take on the characteristic tufted pattern of aggressive wounding), the animal was otherwise in good health, the fur where present was in good condition, and no other cause for the fur lesion could be ascertained. Barbering lesions tend to have sharp edges, with complete or almost complete hair loss with them, in contrast to alopecia, where hair loss is diffuse, incomplete and affects most of the body. These criteria are widely used for diagnosis of barbering [4] without any need for biopsy or histology. Representative pictures of barbering can be found in [4] and [6]. Two cases of ulcerative dermatitis were identified during the study, and because of the severity of the lesions present, the animals were immediately euthanized. Body weight was also recorded every two weeks.

#### Sample collection

Freshly voided urine samples were collected in the afternoon from individual mice for three consecutive days at baseline, 12 weeks and 24 weeks of treatment. For the collection, each mouse was picked-up gently by the tail and placed on top of a wire mouse feeder. The urinary bladder was stimulated by gently massaging the abdomen in a sweeping motion using the free index finger, allowing the mouse to void urine freely into a microcentrifuge tube that was held under the genital area. The samples were kept on ice during collection and then frozen at -80°C until further analysis.

At the end of the experiment (24 weeks), the animals were euthanized by carbon-dioxide. Blood samples were collected in heparinized tubes (BD Vacutainer^®^ Plasma Tubes) and stored in an ice bath. The samples were then centrifuged for 10 minutes(RCF = 1500 x g), and the plasma was aspirated and stored in centrifuge tubes at -80°C until further analysis.

Oxidative stress was determined by measuring the total antioxidant capacity of the urine (TAC), urinary oxidatively modified DNA (8-hydroxy-2deoxyguanosine, 8-OHdG) concentration, plasma total glutathione (GSH) concentration, and plasma oxidized glutathione (GSSG) concentration.

#### Measurement of total antioxidant capacity

The total antioxidant capacity (TAC) of the urine was measured using a commercial kit (#709001, Cayman Chemical, Ann Arbor, MI, USA) according to the manufacturer’s instructions. The assay is based on the ability of the sample to inhibit oxidation of 2, 2′-azino-di-[3-ethylbenzthiazoline sulfonate] to ABTS^+^ by metmyoglobin. The antioxidants in the sample cause a decrease of the absorbance at 405 nm and those values are compared with that of Trolox, a water soluble tocopherol analogue, and is quantified as millimolar Trolox equivalents. The urine samples were diluted 1:40 with buffer before assaying. The results were standardized by urinary creatinine levels using a EIA commercial kit (#500701, Cayman Chemical), following the manufacturer's instructions.

#### Measurement of 8-OHdG

The urinary excretion of 8-OHdG was evaluated using a competitive enzyme-linked immunosorbent assay (ELISA) kit (#589320, Cayman Chemical, Ann Arbor, MI, USA), according to the manufacturer's instructions. The samples were incubated for 1 hour with monoclonal antibody against 8-OHdG in a microtiter plate precoated with 8-OHdG. The final color was developed by the addition of 3, 3, 5, 5-tetramethylbenzidine, and absorbance was measured at 450 nm. The samples were diluted 1:400 with EIA buffer before assaying. The results were standardized by urinary creatinine levels using a commercial kit (#5007, Cayman Chemical), following the manufacturer's instructions.

#### Measurement of GSH and GSSG content

Plasma samples were mixed with an equal volume of cold 5% aqueous 5-sulfo-salicylic acid dihydrate (Sigma-Aldrich, St. Louis, MO, USA) solution to remove protein. The samples were incubated at 4°C for 10 min followed by centrifugation for 10 min at 14,000 rpm and 4°C. The supernatant was collected and stored at -80°C until analysis. GSH/GSSG concentration was measured using the DetectX^®^ glutathione fluorescent detection kit (#K006, Arbor Assays, Ann Arbor, MI, USA) according to the manufacturer’s instructions. This kit utilizes a nonfluorescent molecule, ThioStar, which covalently binds to the free thiol group on GSH to yield a highly fluorescent product. The fluorescent product is read at 510 nm in a fluorescence plate reader with excitation at 390 nm, and the results are expressed in μM. The samples were diluted 1:2.5 with assay buffer and 1:10 with sample diluent to produce a final dilution of 1:50 before assaying.

### Data analysis

All analyses were performed in JMP10 for Windows. Continuous outcome variables [e.g. biomarker levels] were analyzed using Least-Squares General Linear Models (LS-GLM), with suitable transformations applied as needed to meet the assumptions of the methods (i.e. homogeneity of variance, normality of error, and linearity). Categorical outcomes (e.g. Barber status) were analyzed using logistic regression. Logistic regression may overestimate significance in smaller data sets due to overspecification (if too many predictors are included in the model, they appear significant merely because they begin to identify particular individuals). We avoided this problem in two ways. First we took the conservative precaution of performing these analyses as a REML Generalized Linear Model using Firth Bias-adjusted Estimates (which protects against false discovery from overspecification or overdispersion) (REML-GLIM). Second, we simplified the model in each analysis to the minimum number of predictors required while still testing our hypotheses. In particular, initial analyses consistently found no effect of the high-glycemic index “barbering” diet given to half of the non-barbers. Therefore we performed all our final analyses without this variable. Similarly, whenever possible we excluded cage from the model, not just to limit overspecification, but more importantly, as we are primarily interested in discovering biomarkers with potential to translate to human applications we ideally want to find biomarkers that predict behavior without having to include cage as a proxy control for environmental variation (for example if we were screening humans, we would be testing a single patient, and so we wouldn’t have the equivalent of a cage-matched control). Indeed the ideal biomarker would capture the impact of environmental variation at a cage level on the animal’s biology, and so including cage would selectively make the most interesting biomarkers non-significant under Firth Bias-adjusted Estimates and Likelihood Ratio effect tests (because the effect of the biomarker would be correlated with cage).

In all cases except one, animals that developed ulcerative dermatitis were also barbers. Thus, given our earlier findings suggesting that these behaviors are essentially different behavioral expressions of the same disease process [7,37] we therefore treated both behaviors as a single outcome.

#### Is there evidence of oxidative stress in barbers at baseline?

To test whether oxidative stress biomarkers predicted Barbering/ulcerative dermatitis status at baseline, we performed a REML-GLIM analysis, testing whether total antioxidant capacity and 8-OHdG could predict barbering status, blocking by cage. For 6 animals, biomarker data could not be accurately determined and were eliminated from the dataset. N = 26 animals.

#### Does NAC cure or prevent barbering?

To investigate the effect of treatment on Barbering/ulcerative dermatitis status at 24 weeks, we performed a REML-GLIM analysis testing the effects of initial barber status, NAC treatment, and their interaction. Two mice were euthanized for ulcerative dermatitis earlier in the experiment but were still included in the analysis as positive for Barbering/ulcerative dermatitis. N = 32 animals.

#### Is there evidence of oxidative stress in barbers at 24 weeks?

If oxidative stress is a biomarker of disease mechanism, then the same relationships should be observed prior to treatment. If NAC works by manipulating the biomarkers, then NAC and control animals should show the same relationship; whereas if NAC simply overrides a biomarker’s effect without changing it, then an interaction between NAC and the biomarker should be observed. Therefore, we analyzed whether biomarkers at 24 weeks predicted final outcome using a REML-GLIM analysis, testing whether total antioxidant capacity, 8-OHdG, GSSG and GSH could predict barbering status, while NAC treatment and initial barbering status were included as controls. We initially tested for possible interactions, such as if the effect of GSH depends on NAC, but did not find any interactions. Therefore, the model was simplified to main effects only. Biomarker data for four animals (including two euthanized prior to 24 weeks) could not be determined and therefore excluded from the dataset. N = 28 animals.

#### Is there evidence that high 8-OHdG is a response to oxidative stress?

As 8-OHdG showed a complex series of responses, we tested the interpretation of urinary 8-OHdG levels as response to oxidative stress with a LS-GLM, which predicted 8-OHdG levels given the value of the other biomarkers (controlling for NAC and barbering status). N = 28 animals.

#### Is there evidence that NAC treatment influences biomarkers?

Given that oxidative stress biomarkers predict barbering status both before and after treatment, we tested whether NAC, initial barbering status, and their interaction predicted final biomarker levels using a LS-GLM. N = 28 animals.

#### Is there evidence that oxidative stress biomarkers predict treatment response?

Finally we tested whether oxidative stress biomarkers prior to treatment predicted treatment efficacy. Initially, total antioxidant capacity and 8-OHdG in the same model were tested, but there was evidence of co-linearity. Therefore each biomarker was tested separately in a REML-GLIM predicting final Barbering/ulcerative dermatitis status given NAC treatment, biomarker levels, and their interaction; and controlling for the initial Barbering/ulcerative dermatitis status and its interaction with NAC. N = 32 animals for 8-OHdG; N = 26 for total antioxidant capacity.

## Results

### Is there evidence of oxidative stress in barbers?

**Fig 1:**
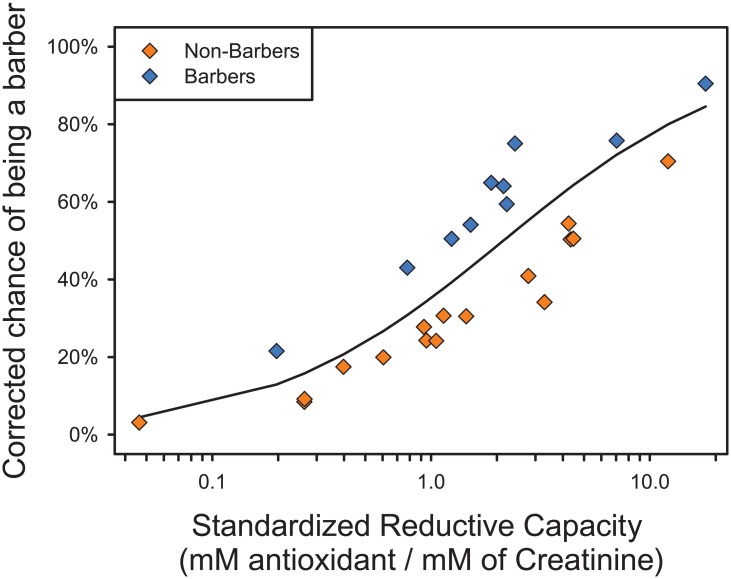
Total antioxidant capacity (standardized reductive capacity—mM Trolox/mM creatinine) predicts barbering status at baseline. Total antioxidant capacity measures the activation of antioxidant defenses. All but two barbering animals showed total antioxidant capacity above the midpoint of the range observed. To show the effect of including cage as a control variable in the model, data are plotted as the observed value (0 or 100%) corrected for cage and 8-OhdG levels. (Orange squares: animals classified as non-barbers at the beginning of the study. Blue squares: animals classified as barbers at the beginning of the study).

Animals with higher total antioxidant capacity at baseline were significantly more likely to be barbers (LR Chi-Square = 4.486; P = 0.0342) (Fig 1). Baseline 8-OHdG levels did not predict barbering at baseline (LR Chi-Square < 0.001; P > 0.9999).

### Does NAC cure or prevent barbering?

**Fig 2:**
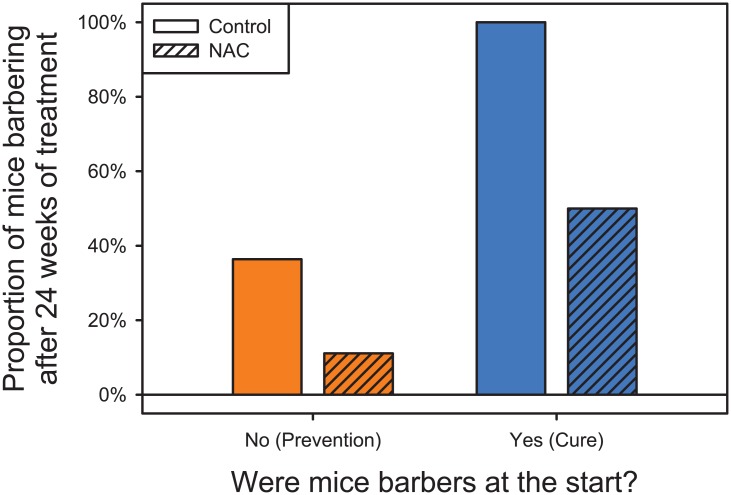
The effect of initial barber status and NAC treatment on final barber status. NAC treatment significantly reduced the risk of barbering after 24 weeks of treatment, both preventing onset in healthy animals, and curing animals with the behavior. (Orange bars: animals classified as non-barbers at the beginning of the study. Blue bars: animals classified as barbers at the beginning of the study. Open bars: control mice. Hashed bars: NAC treated mice).

Animals that were barbers at the start of the experiment were significantly more likely to be barbers at the end (LR Chi-Square = 8.566; P = 0.0034); and mice receiving NAC were significantly less likely to be barbers by the end of the experiment (LR Chi-Square = 5.063; P = 0.0244). There was no evidence of an interaction between NAC and initial barbering status (LR Chi-Square = 0.2694; P = 0.6037). Thus there was no evidence that the effect of NAC differed between healthy animals (i.e. prevention) and affected animals (i.e. cure) (Fig 2).

### Is there evidence of oxidative stress in barbers at 24 weeks?

None of the biomarkers of oxidative stress at 24 weeks showed a significant interaction with NAC or baseline barber status (all tests P>0.05). Instead we observed that these response curves were shifted by NAC or baseline status such that the animals with the potential to change (i.e. NAC treated barbers that stop barbering; or control non-barbers that displayed barbering behavior later) were predicted by biomarkers at 24 weeks in a consistent manner. Thus, animals that were barbers at the start of the experiment were significantly more likely to be barbers at the end (LR Chi-Square = 10.78; P = 0.0010); and mice receiving NAC were significantly less likely to be barbers by the end of the experiment (LR Chi-Square = 9.021; P = 0.0027). Note that including biomarkers in the analysis improves the predictive power of NAC treatment 10 fold. Animals with higher total antioxidant capacity at the end of the experiment were significantly more likely to be barbers at this time point (LR Chi-Square = 5.562; P = 0.0183). Similarly, the risk of barbering/ulcerative dermatitis increased with decreasing 8-OHdG levels (LR Chi-Square = 4.174; P = 0.0410), and with increasing GSH (LR Chi-Square = 10.45; P = 0.0012) and GSSG (LR Chi-Square = 4.382; P = 0.0363) levels.

### Is there evidence that high 8-OHdG is a response to oxidative stress?

The only predictor of 8-OHdG at 24 weeks was GSSG, which showed a positive correlation (F_1,22_ = 7.2862; P = 0.0131; r = 0.50). Neither GSH, nor total antioxidant capacity predicted 8-OHdG once the predictive power of GSSG was taken into account. This result was consistent whether or not barbering and NAC were included as controlling variables.

### Is there evidence that NAC treatment influences biomarkers?

**Fig 3:**
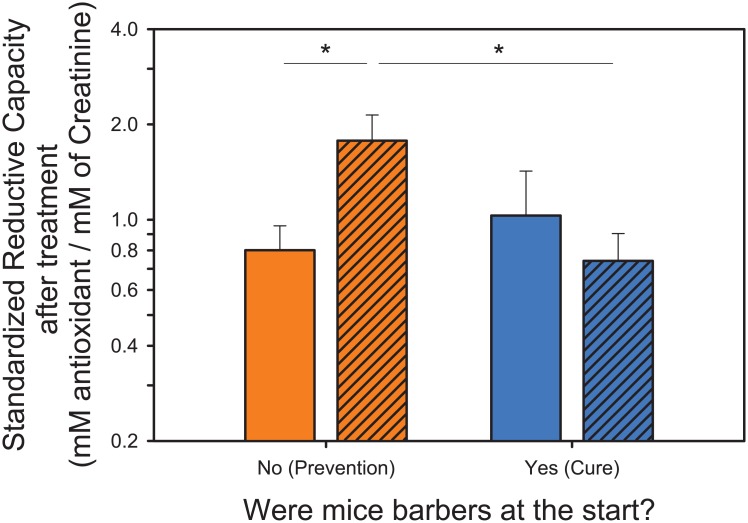
Levels of total antioxidant capacity (standardized reductive capacity—mM Trolox/mM creatinine) of non-barbers and barbers at baseline, after NAC treatment. NAC treated non-barbers showed higher total antioxidant capacity than control non-barbers, or NAC treated barbers at the end of the study. (Orange bars: animals classified as non-barbers at the beginning of the study. Blue bars: animals classified as barbers at the beginning of the study. Open bars: control mice. Hashed bars: NAC treated mice).

8-OHdG, GSH and GSSG levels at 24 weeks were unaffected by NAC treatment, initial barber status or their interaction (all tests, P < 0.05). However, total antioxidant capacity was significantly predicted by the interaction of initial barber status and NAC treatment (F_1,26_ = 6.0337; P = 0.0210), whereby NAC treated non-barbers showed higher total antioxidant capacity than control non-barbers, or NAC treated barbers (Fig 3).

### Is there evidence that oxidative stress biomarkers predict treatment response?

Neither total antioxidant capacity at baseline, nor its interaction with NAC treatment predicted final outcome in our planned analyses. The levels of urinary 8-OHdG were also not predictive of final barber/ulcerative dermatitis status (all tests P>0.05). However, this was likely a power issue. In both cases, we saw some evidence that both biomarkers might predict which non-barbering control animals became ill, and we could find models where these effects were significant. However, as these were not part of our planned analysis, and these results were not robust (i.e. they only held up in a limited number of models), and did not reflect our research question, we have chosen not to report them to avoid the risk of false discovery and p-hacking.

## Discussion

Our primary objective in this study was to establish the association between oxidative stress and barbering in mice and to determine whether antioxidant therapy could reverse and/or prevent the occurrence of hair-pulling behavior. There is growing evidence that chronic oxidative stress damage can cause several pathological conditions, including cardiovascular disease [38], cancer [25,39,40], diabetes [41], neurological and psychiatric disorders [29,31,42,43]. Although it has been shown that levels of antioxidants and oxidative stress biomarkers are altered in those disorders, the pathophysiology is not completely understood [44].

Oxidative stress is the result of an imbalance between ROS and antioxidant defenses. The level of antioxidants in the body is regulated by redox-sensitive regulatory molecules in the cell, triggering homeostatic responses to prevent cellular injury [45]. The total antioxidant capacity represents the sum of the antioxidant capacity of all antioxidants present in a biological sample and can be useful to evaluate how the organism could respond to an oxidative insult [46,47]. In this study, we observed that animals with higher antioxidant capacity at the start of the experiment were significantly more likely to be barbers. Under oxidative stress, cells must increase their antioxidant capacity to counterbalance the higher ROS production and maintain homeostasis [48]. ROS can activate transcription of oxidative stress-inducible genes *in vitro* [49]; however the complete network of regulators of ROS responses that activate such genes remains unclear [22]. High levels of oxidative stress can increase total antioxidant status in diabetic patients [50,51], soccer players [21], and glaucoma patients [52]. In chickens [*Gallus gallus domesticus*], short and chronic supplementation of stress hormones (which are inducers of pro-oxidant production) led to an increase of plasma antioxidant capacity [53]. Therefore in our study, the increased antioxidant capacity observed in hair-pullers is best interpreted as an adaptive response to oxidative stress [54,55]. This interpretation is further supported by the full panel of biomarkers taken at the end of the study, as discussed below.

In this study, we evaluated whether antioxidant therapy could cure barbering behavior in C57BL/6J mice. The fact that mice receiving NAC were significantly less likely to be barbers suggests that NAC is effective in the treatment of compulsive hair-pulling in mice. These results are concordant with the previous research examining the efficiency of NAC in hair-pulling behavior in humans. In that study, 56% of patients with TTM, receiving NAC for 12 weeks in a double-blind placebo trial (N = 50), had a reduction in hair-pulling symptoms compared to 16% of patients taking placebo [9]. Furthermore, the evidence that the effect of NAC was the same between healthy animals and affected animals, suggests that NAC could be useful not only to cure but also to prevent barbering behavior.

NAC is a source of cysteine and a rate-limiting precursor of GSH, the brain’s primary antioxidant [56]. The fact that NAC treatment increased levels of urinary antioxidants in non-barbers supports the hypothesis that administration of NAC could restore GSH levels in the blood and brain [57,58] and increase the body total antioxidant capacity [59]. In support of this, NAC treatment successfully prevented GSH depletion in rat models of oxidative stress [13,33]. The evidence that NAC treated non-barbers showed higher total antioxidant capacity than NAC treated barbers suggests that although NAC improved the GSH synthesis in both groups, this increase was not reflected in the urinary antioxidant capacity of barbers because of their higher levels of ROS and consequent higher usage of antioxidants.

This interpretation is supported by the full panel of biomarkers taken at 24 weeks. Thus regardless of initial barbering/ulcerative dermatitis status and NAC treatment, higher urinary total antioxidant capacity, higher plasma GSH and higher plasma GSSG all independently and additively predicted a higher probability of a mouse being a barber at 24 weeks. This is consistent with oxidative stress having led to a wholescale activation of antioxidant defenses in these animals. From this perspective it might seem contradictory that lower urinary 8-OHdG also predicted a higher risk of barbering. This result warrants additional discussion (see below), but in brief, we believe this result is highly informative, as it potentially differentiates the mice that are succeeding (high 8-OHdG) *versus* failing to repair DNA in response to damage. Thus not only do we detect biomarkers of elevated oxidative stress in barbering mice, but we observe that the mice that are potentially coping with this stress (in terms of DNA repair) are protected.

Although being the most widely used biomarker to assess oxidative DNA damage, the interpretation of urinary 8-OHdG levels is ambiguous [28]. Some studies have shown higher 8-OHdG levels in patients with hypertension [60], depression [61], smokers [62], workers exposed to diesel fumes, asbestos and heavy metals [27] and cancer patients [63], which was interpreted as an indication of oxidative DNA damage. However, 8-OHdG levels comprise a combination of ROS production, cellular redox status, antioxidant defense mechanisms, and DNA repair systems, not simply oxidative DNA damage [64,65]. They represent a dynamic balance between rates of oxidative DNA damage and rates of repair of that damage [28]. 8-OHdG is excreted in urine only after DNA damage repair [66], usually within 24 hours [67]. In addition, an increase in urinary 8-OHdG levels is correlated with a decrease in intracellular 8-OHdG, further emphasizing the acute and eliminative context of this biomarker [66].

Thus low excretion of urinary 8-OHdG in barbers at 24 weeks may reflect two possible mechanism. First it may indicate animals that are failing to cope with oxidative stress and successfully repair DNA. Or, second, it may reflect a deficient repair capacity and an accumulation of 8-OHdG in DNA. For instance, patients with normal tension glaucoma have higher serum total antioxidant capacity and lower levels of urinary 8-OHdG compared to healthy controls [52]. Either interpretation is consistent with the overall interpretation that elevated oxidative stress is a causal mechanism in barbering, and that NAC is targeting this mechanism directly.

Nevertheless, the final result in the full biomarker panel supports the first of these two possible explanations. The only predictor of 8-OHdG levels at 24 weeks was plasma GSSG, which showed a positive correlation. Since GSSG is the oxidized form of GSH [68], it is expected that with an increase in oxidative processes, an increase in oxidative damage/repair may also be observed. Thus, given that high GSSG predicts high 8-OHdG it is unlikely that DNA repair mechanisms are inherently deficient in these animals; and the fact that high GSSG and low 8-OHdG predict barbering is consistent with high 8-OHdG identifying the animals that are coping in terms of DNA repair despite high oxidative stress levels.

Thus in summary of the biomarker data, regardless of treatment, animals that were barbers at the end differed consistently from those that were not (bearing in mind that this included barbers that were cured, and control animals that became barbers). This is an ideal signal in a biomarker study–that the biomarkers predict outcome regardless of treatment. A very famous example of this comes from the OCD literature, where changes in fMRI indicate how well each patient responded to treatment regardless of whether the treatment was behavioral or pharmacological [69]. Furthermore, the lack of effect of NAC on GSH, GSSG, or 8-OHdG overall makes perfect sense given the fact that health animals treated with NAC show increases in TAC–animals that do not need the extra NAC excrete it rather than produce additional GSH.

To our knowledge, this is the first evidence of an association between oxidative stress and compulsive hair-pulling behavior in mice. We recognized the limitation of using urine and blood samples to measure the levels of biomarkers of oxidative stress, as the oxidative damage is not occurring directly in those tissues. Whereas the oxidative process could be taking place in the brain, it also could be occurring in any part of the body. Future investigations focusing on the relationship between brain biomarkers of oxidative stress and barbering behavior are required to elucidate these mechanisms. Nevertheless, these results suggest a potential alternative therapy. Thus if oxidative stress in the brain is the key causal mechanism, then intranasal administration of GSH should also effectively treat the behavior, and might circumvent the clinical issues with NAC. Following the experiment reported here, we recently tested this hypothesis by comparing the efficacy of oral NAC and intranasal GSH to treat ulcerative dermatitis, and found that as predicted, intranasal GSH was highly effective [70].

## Conclusion

In conclusion, the results from the present study suggest that barbering behavior is associated with oxidative stress and the high antioxidant capacity in barbers may result from up-regulated antioxidant production in response to oxidative stress. NAC is effective in preventing and/or curing barbering probably through the improvement of GSH synthesis in the brain, thereby preventing oxidative damage. Animals with markers of elevated oxidative stress defenses are more likely to be barbers; and animals with decreasing levels of 8-OHdG, and possibly low DNA repair, are more likely to be barbers. Our experiment did not find compelling evidence that urinary total antioxidant capacity or urinary 8-OHdG could predict response to NAC treatment.

## Supporting information

S1 Data All original data and analyses are provided as SAS code. (DOCX)
