# Supplementary material for: Preventing, treating, and predicting barbering: A fundamental role for biomarkers of oxidative stress in a mouse model of Trichotillomania
Source: PLoS One. 2017 Apr 20;12(4):e0175222. doi: 10.1371/journal.pone.0175222 (PMC5398524; doi:10.1371/journal.pone.0175222)
Supplement: S1 Data — (DOCX) [file pone.0175222.s001.docx]

# Supplementary data

The raw data for each of the analyses are presented below. Each data set is given as a SAS code for the data itself, and the equivalent analysis to that performed in JMP (and reported in the text). Data are presented in SAS format as this is a simple text format. The data and code were generated as direct exports from JMP, and additional SAS code added as needed (for instance, JMP does not export code for post-hoc tests).

Note, however, that SAS rounds to less precision than JMP, and can give slightly different results, especially for REML and GLIM methods. In particular, SAS does not have native procedures allowing both Firth correction and Likelihood Ratio tests for a logistic regression. As a result the GENMOD analyses are less conservative, producing more significant results than the conservative Firth corrected results generated by JMP which we report in the paper. Firth correction also allows the analysis of a model that would otherwise be over-specified or show quasi-complete separation. In these cases the raw data is provided, but not the equivalent SAS code.

# Is there evidence of oxidative stress in barbers?

There is no equivalent analysis in SAS to a Firth corrected Likelihood Ratio tested model, which for this analysis is required to prevent overspecification introduced by stratifying by cage. Thus only the raw data are provided.

**DATA** Full_physiology_20151028_paper; INPUT Cage Baseline_BarberorUD &$ Baseline_log_r_c_Bas Baseline_OHdG_creat_ng_mg_; Lines;

**1** N -**1.075309656** **366.2476023**

**1** N -**1.271745824** **290.9366475**

**1** Y **0.20135138** **519.171116**

**2** Y -**1.759909341** **396.1165091**

**2** Y -**0.20565529** **121.3204449**

**3** N -**1.632247031** **1132.502974**

**3** N -**0.892838445** **175.5884011**

**4** N **.** **385.5628334**

**4** Y -**0.722362181** **350.8399619**

**5** Y -**0.707817046** **263.7554486**

**5** Y -**1.162532492** **279.2804741**

**5** Y **.** **372.0680347**

**6** Y -**0.872849254** **234.584424**

**6** Y -**0.959795219** **287.8971272**

**7** N **0.029530525** **293.5106096**

**7** N -**0.424858274** **389.2674209**

**8** Y -**0.7791013** **229.3375072**

**8** N -**2.387698655** **405.3226959**

**8** N -**1.632996523** **347.8199664**

**9** Y **.** **238.5031435**

**9** N -**1.030200328** **113.7186694**

**10** N -**1.086239671** **267.6629858**

**10** N -**0.402847409** **326.3572422**

**11** Y -**0.67040959** **380.9649549**

**11** N -**0.53500169** **250.0186991**

**12** N -**0.415577172** **325.8199641**

**12** N -**1.454016707** **288.5257224**

**12** N -**0.996677117** **286.937991**

**13** N **.** **470.97563**

**13** N **.** **397.7962769**

**14** N -**0.609230908** **263.320211**

**14** N **.** **358.9976382**

;

**RUN**;

# Does NAC cure or prevent barbering?

This analysis is equivalent to performing the same analysis in JMP without the more conservative Firth correction.

**DATA** Full_physiology_20151028_paper; INPUT NAC &$ Baseline_BarberorUD &$ _24_weeks_BarberorUD &$; Lines;

Control N N

Control N Y

Control Y Y

NAC Y Y

NAC Y Y

Control N Y

Control N N

Control N Y

Control Y Y

NAC Y N

NAC Y N

NAC Y N

NAC Y Y

NAC Y Y

NAC N N

NAC N N

NAC Y N

NAC N N

NAC N N

Control Y Y

Control N N

NAC N N

NAC N Y

Control Y Y

Control N N

Control N N

Control N N

NAC N N

NAC N N

NAC N N

Control N N

Control N Y

;

**RUN**;

**PROC** **GENMOD** DATA=Full_physiology_20151028_paper desc;

CLASS NAC Baseline_BarberorUD;

MODEL _24_weeks_BarberorUD = Baseline_BarberorUD NAC Baseline_BarberorUD*NAC/ DIST=Binomial LINK=Logit ALPHA=**0.05** type3;

LSMEANS Baseline_BarberorUD*NAC/ ilink;

**RUN**;

# Is there evidence of oxidative stress in barbers at 24 weeks?

This analysis is equivalent to performing the same analysis in JMP without the more conservative Firth correction.

**DATA** Full_physiology_20151028_paper; INPUT NAC &$ Baseline_BarberorUD &$ _24_weeks_BarberorUD &$ _24_weeks_log_r_c_24_w _24_weeks_log_OHdG_creat_ _24_weeks_Free_GSH_uM _24_weeks_GSSG; Lines;

Control N N -**1.226698515** **1.499412126** **9.744617672** **21.7042812**

Control N Y -**1.275237629** **1.632659713** **11.6756624** **46.29722435**

Control Y Y -**1.221965926** **1.25163822** **9.923418109** **42.03977258**

NAC Y Y -**1.190860776** **1.26245109** **11.74718257** **39.92444598**

NAC Y Y -**1.356033351** **1.757244151** **8.17117382** **75.5098812**

Control N Y -**1.504044775** **1.644044493** **17.64759702** **32.7411442**

Control N N -**1.492622119** **1.552424846** **8.278454083** **37.16482913**

Control N Y **.** **.** **.** **.**

Control Y Y **.** **.** **.** **.**

NAC Y N -**1.343735978** **1.219060332** **9.029415921** **21.62426594**

NAC Y N -**1.164222742** **1.404320467** **8.02813347** **26.046621**

NAC Y N -**1.106340716** **1.871280973** **7.992373382** **37.82964256**

NAC Y Y -**1.100724507** **1.39252109** **7.74205277** **18.4724691**

NAC Y Y -**0.993969564** **1.34849957** **10.60285977** **39.06593924**

NAC N N -**0.80711978** **1.650598898** **8.206933908** **43.72275279**

NAC N N -**0.563075517** **1.572987708** **9.208216359** **42.53202458**

NAC Y N -**1.211580979** **1.384890797** **6.883810669** **41.22001311**

NAC N N -**1.379897434** **1.620448385** **7.920853207** **58.43336106**

NAC N N -**0.775236195** **1.282622113** **7.134131281** **15.6962855**

Control Y Y -**0.956747615** **1.517987203** **8.385734345** **16.93876824**

Control N N -**1.456545956** **1.188084374** **6.597729968** **16.7303144**

NAC N N -**0.78964891** **1.505149978** **7.813572945** **17.92335162**

NAC N Y -**0.685515822** **1.438700533** **12.03326327** **28.84100221**

Control Y Y -**0.942273891** **1.534533756** **6.418929531** **26.11064182**

Control N N -**1.321883943** **1.223755454** **.** **.**

Control N N -**1.020388666** **1.606381365** **8.17117382** **35.52691458**

Control N N -**0.684621406** **1.595936906** **7.241411544** **.**

NAC N N -**0.659348414** **1.804752602** **8.063893557** **14.38141917**

NAC N N -**0.702040631** **1.315550534** **7.420211982** **17.34578817**

NAC N N -**0.871775403** **1.605412798** **6.454689618** **18.81318566**

Control N N -**0.669919633** **1.679972694** **7.563252332** **28.29041266**

Control N Y -**0.845636122** **1.191451014** **5.918288305** **21.13481588**

;

**RUN**;

**PROC** **GENMOD** DATA=Full_physiology_20151028_paper desc;

CLASS NAC Baseline_BarberorUD;

MODEL _24_weeks_BarberorUD = NAC Baseline_BarberorUD _24_weeks_log_r_c_24_w _24_weeks_log_OHdG_creat_ _24_weeks_Free_GSH_uM _24_weeks_GSSG/ DIST=Binomial LINK=Logit Type3;

**RUN**;

# Is there evidence that high 8-OHdG is a response to oxidative stress?

This analysis is directly equivalent to performing the same analysis in JMP. Partial correlation coefficients reported were calculated by hand.

**DATA** Full_physiology_20151028_paper; INPUT NAC &$ _24_weeks_BarberorUD &$ _24_weeks_log_r_c_24_w _24_weeks_log_OHdG_creat_ _24_weeks_Free_GSH_uM _24_weeks_GSSG; Lines;

Control N -**1.226698515** **1.499412126** **9.744617672** **21.7042812**

Control Y -**1.275237629** **1.632659713** **11.6756624** **46.29722435**

Control Y -**1.221965926** **1.25163822** **9.923418109** **42.03977258**

NAC Y -**1.190860776** **1.26245109** **11.74718257** **39.92444598**

NAC Y -**1.356033351** **1.757244151** **8.17117382** **75.5098812**

Control Y -**1.504044775** **1.644044493** **17.64759702** **32.7411442**

Control N -**1.492622119** **1.552424846** **8.278454083** **37.16482913**

Control Y **.** **.** **.** **.**

Control Y **.** **.** **.** **.**

NAC N -**1.343735978** **1.219060332** **9.029415921** **21.62426594**

NAC N -**1.164222742** **1.404320467** **8.02813347** **26.046621**

NAC N -**1.106340716** **1.871280973** **7.992373382** **37.82964256**

NAC Y -**1.100724507** **1.39252109** **7.74205277** **18.4724691**

NAC Y -**0.993969564** **1.34849957** **10.60285977** **39.06593924**

NAC N -**0.80711978** **1.650598898** **8.206933908** **43.72275279**

NAC N -**0.563075517** **1.572987708** **9.208216359** **42.53202458**

NAC N -**1.211580979** **1.384890797** **6.883810669** **41.22001311**

NAC N -**1.379897434** **1.620448385** **7.920853207** **58.43336106**

NAC N -**0.775236195** **1.282622113** **7.134131281** **15.6962855**

Control Y -**0.956747615** **1.517987203** **8.385734345** **16.93876824**

Control N -**1.456545956** **1.188084374** **6.597729968** **16.7303144**

NAC N -**0.78964891** **1.505149978** **7.813572945** **17.92335162**

NAC Y -**0.685515822** **1.438700533** **12.03326327** **28.84100221**

Control Y -**0.942273891** **1.534533756** **6.418929531** **26.11064182**

Control N -**1.321883943** **1.223755454** **.** **.**

Control N -**1.020388666** **1.606381365** **8.17117382** **35.52691458**

Control N -**0.684621406** **1.595936906** **7.241411544** **.**

NAC N -**0.659348414** **1.804752602** **8.063893557** **14.38141917**

NAC N -**0.702040631** **1.315550534** **7.420211982** **17.34578817**

NAC N -**0.871775403** **1.605412798** **6.454689618** **18.81318566**

Control N -**0.669919633** **1.679972694** **7.563252332** **28.29041266**

Control Y -**0.845636122** **1.191451014** **5.918288305** **21.13481588**

;

**RUN**;

**PROC** **GLM** DATA=Full_physiology_20151028_paper ALPHA=**0.05**;

CLASS NAC _24_weeks_BarberorUD;

MODEL _24_weeks_log_OHdG_creat_ = _24_weeks_Free_GSH_uM _24_weeks_GSSG NAC _24_weeks_BarberorUD _24_weeks_log_r_c_24_w/ss3 solution;

**RUN**;

# Is there evidence that NAC treatment influences biomarkers?

This analysis is directly equivalent to performing the same analysis in JMP. Least-sqaures means and posthoc tests are only performed for the significant interaction seen for Total Redcutive Capacity (the first of the analyses).

**DATA** Full_physiology_20151028_paper; INPUT NAC &$ Baseline_BarberorUD &$ _24_weeks_log_r_c_24_w _24_weeks_log_OHdG_creat_ _24_weeks_Free_GSH_uM _24_weeks_GSSG; Lines;

Control N -**1.226698515** **1.499412126** **9.744617672** **21.7042812**

Control N -**1.275237629** **1.632659713** **11.6756624** **46.29722435**

Control Y -**1.221965926** **1.25163822** **9.923418109** **42.03977258**

NAC Y -**1.190860776** **1.26245109** **11.74718257** **39.92444598**

NAC Y -**1.356033351** **1.757244151** **8.17117382** **75.5098812**

Control N -**1.504044775** **1.644044493** **17.64759702** **32.7411442**

Control N -**1.492622119** **1.552424846** **8.278454083** **37.16482913**

Control N **.** **.** **.** **.**

Control Y **.** **.** **.** **.**

NAC Y -**1.343735978** **1.219060332** **9.029415921** **21.62426594**

NAC Y -**1.164222742** **1.404320467** **8.02813347** **26.046621**

NAC Y -**1.106340716** **1.871280973** **7.992373382** **37.82964256**

NAC Y -**1.100724507** **1.39252109** **7.74205277** **18.4724691**

NAC Y -**0.993969564** **1.34849957** **10.60285977** **39.06593924**

NAC N -**0.80711978** **1.650598898** **8.206933908** **43.72275279**

NAC N -**0.563075517** **1.572987708** **9.208216359** **42.53202458**

NAC Y -**1.211580979** **1.384890797** **6.883810669** **41.22001311**

NAC N -**1.379897434** **1.620448385** **7.920853207** **58.43336106**

NAC N -**0.775236195** **1.282622113** **7.134131281** **15.6962855**

Control Y -**0.956747615** **1.517987203** **8.385734345** **16.93876824**

Control N -**1.456545956** **1.188084374** **6.597729968** **16.7303144**

NAC N -**0.78964891** **1.505149978** **7.813572945** **17.92335162**

NAC N -**0.685515822** **1.438700533** **12.03326327** **28.84100221**

Control Y -**0.942273891** **1.534533756** **6.418929531** **26.11064182**

Control N -**1.321883943** **1.223755454** **.** **.**

Control N -**1.020388666** **1.606381365** **8.17117382** **35.52691458**

Control N -**0.684621406** **1.595936906** **7.241411544** **.**

NAC N -**0.659348414** **1.804752602** **8.063893557** **14.38141917**

NAC N -**0.702040631** **1.315550534** **7.420211982** **17.34578817**

NAC N -**0.871775403** **1.605412798** **6.454689618** **18.81318566**

Control N -**0.669919633** **1.679972694** **7.563252332** **28.29041266**

Control N -**0.845636122** **1.191451014** **5.918288305** **21.13481588**

;

**RUN**;

**PROC** **GLM** DATA=Full_physiology_20151028_paper ALPHA=**0.05**;

CLASS NAC Baseline_BarberorUD;

MODEL _24_weeks_log_r_c_24_w = NAC Baseline_BarberorUD Baseline_BarberorUD*NAC/ss3;

Lsmeans Baseline_BarberorUD*NAC/ stderr plots=none slice=nac slice=Baseline_BarberorUD ;

**RUN**;

**PROC** **GLM** DATA=Full_physiology_20151028_paper ALPHA=**0.05**;

CLASS NAC Baseline_BarberorUD;

MODEL _24_weeks_log_OHdG_creat_ = NAC Baseline_BarberorUD Baseline_BarberorUD*NAC/ss3;

**RUN**;

**PROC** **GLM** DATA=Full_physiology_20151028_paper ALPHA=**0.05**;

CLASS NAC Baseline_BarberorUD;

MODEL _24_weeks_Free_GSH_uM = NAC Baseline_BarberorUD Baseline_BarberorUD*NAC/ss3;

**RUN**;

**PROC** **GLM** DATA=Full_physiology_20151028_paper ALPHA=**0.05**;

CLASS NAC Baseline_BarberorUD;

MODEL _24_weeks_GSSG = NAC Baseline_BarberorUD Baseline_BarberorUD*NAC/ss3;

**RUN**;

# Is there evidence that oxidative stress biomarkers predict treatment response?

There is no equivalent analysis in SAS to a Firth corrected Likelihood Ratio tested model, which for these analyses is required to overcome quasi-complete separation of the model. Thus only the raw data is provided.

**DATA** Full_physiology_20151028_paper; INPUT NAC &$ Baseline_BarberorUD &$ Baseline_log_r_c_Bas _24_weeks_BarberorUD &$ log_baseline_ohdg_creat; Lines;

Control N -**1.075309656** N **2.56377479019141**

Control N -**1.271745824** Y **2.46379843009744**

Control Y **0.20135138** Y **2.71531052256205**

NAC Y -**1.759909341** Y **2.59782294303954**

NAC Y -**0.20565529** Y **2.08393399426359**

Control N -**1.632247031** Y **3.05403935116177**

Control N -**0.892838445** N **2.24449582418603**

Control N **.** Y **2.58609516312696**

Control Y -**0.722362181** Y **2.5451090551735**

NAC Y -**0.707817046** N **2.42120143995493**

NAC Y -**1.162532492** N **2.4460405730549**

NAC Y **.** N **2.57062236029372**

NAC Y -**0.872849254** Y **2.3702991723344**

NAC Y -**0.959795219** Y **2.45923733126428**

NAC N **0.029530525** N **2.46762380441653**

NAC N -**0.424858274** N **2.59024805767289**

NAC Y -**0.7791013** N **2.36047508760349**

NAC N -**2.387698655** N **2.6078009225874**

NAC N -**1.632996523** N **2.54135450880083**

Control Y **.** Y **2.37749410746715**

Control N -**1.030200328** N **2.05583176945299**

NAC N -**1.086239671** N **2.42758831823869**

NAC N -**0.402847409** Y **2.51369325457635**

Control Y -**0.67040959** Y **2.58088502661114**

Control N -**0.53500169** N **2.39797249112105**

Control N -**0.415577172** N **2.51297769149131**

Control N -**1.454016707** N **2.46018453707029**

NAC N -**0.996677117** N **2.4577880532629**

NAC N **.** N **2.6729984357269**

NAC N **.** N **2.5996607141093**

Control N -**0.609230908** N **2.42048419442285**

Control N **.** Y **2.55509159141932**

;

**RUN**;
